# The Ancient Chinese Decoction Yu-Ping-Feng Suppresses Orthotopic Lewis Lung Cancer Tumor Growth Through Increasing M1 Macrophage Polarization and CD4^+^ T Cell Cytotoxicity

**DOI:** 10.3389/fphar.2019.01333

**Published:** 2019-11-08

**Authors:** Lixin Wang, Wenbin Wu, Xiaowen Zhu, Wanyi Ng, Chenyuan Gong, Chao Yao, Zhongya Ni, Xuewei Yan, Cheng Fang, Shiguo Zhu

**Affiliations:** ^1^Department of Immunology and Pathogenic Biology, School of Basic Medical Sciences, Shanghai University of Traditional Chinese Medicine, Shanghai, China; ^2^Experiment Animal Center, Experiment Center for Science and Technology, Shanghai University of Traditional Chinese Medicine, Shanghai, China; ^3^Laboratory of Integrative Medicine, School of Basic Medical Sciences, Shanghai University of Traditional Chinese Medicine, Shanghai, China

**Keywords:** non-small cell lung cancer, Yu-Ping-Feng, Macrophages, CD4^+^ T cells, tumor microenvironment

## Abstract

**Background:** The tumor microenvironment (TME) has a deep influence on cancer progression and has become into a new target for cancer treatment. In our previous study, we found that Yu-Ping-Feng (YPF), an ancient Chinese herbal decoction, significantly inhibited the Lewis lung cancer (LLC) tumor growth in a subcutaneous xenograft tumor model, and prolonged the survival of tumor-bearing mice. But the regulation of Yu-Ping-Feng on tumor microenvironment is unknown.

**Methods:** To access the effect of Yu-Ping-Feng on non-small cell lung cancer, an orthotopic luciferase stably expressed Lewis lung cancer tumor model was established on C57BL/6 mice, and then the survival and the tumor growth were evaluated. To address the tumor microenvironment immune regulation, the percentages of CD4^+^ T cells, CD8^+^ T cells, natural killer cells (NK), regulatory T cells (Treg), macrophages, and myeloid-derived suppressor cells (MDSC) in spleens and tumor tissues, the macrophage polarization and CD4^+^ T cell cytotocixity were analyzed by flow cytometry, biophotonic cell killing activity assay, real-time PCR and western-blot.

**Results:** Yu-Ping-Feng significantly prolonged orthotopic lung tumor-bearing mouse survival, and increased the percentages of CD4^+^ T cell and M1 macrophages and the cytotoxicity of CD4^+^ T cells. Yu-Ping-Feng significantly enhanced macrophage-mediated lysis of LLC in a concentration-dependent manner, and had no effect on CD4^+^ T cell-mediated lysis of LLC, but significantly increased CD4^+^ T cell-mediated lysis after co-incubated with macrophages. In addition, Yu-Ping-Feng induced M1 macrophage polarization through promoting the phosphorylation of STAT1.

**Conclusion:** Yu-Ping-Feng induced M1 macrophages polarization, and then activated CD4^+^ T lymphocytes, resulting in killing of LLC cells. Yu-Ping-Feng was a potent regulator of M1 macrophage polarization and might have a promising application in tumor immunotherapy.

## Introduction

Lung cancer is the leading cause of cancer-related death in China and is responsible for more than 1 million deaths worldwide annually (Siegel et al., 2015; Chen et al., 2016b). Non-small-cell lung cancer (NSCLC) accounts for approximately 85% of all lung cancer cases and is diagnosed as locally advanced or metastatic at presentation in 70% of patients (Mountzios et al., 2016). A series of therapeutic interventions, such as surgical operation, chemotherapy and radiation therapy have been developed to benefit the treatment of NSCLC (Rafei et al., 2017). However, for advanced stage, the five-year survival rate remains low. Therefore, efficient therapeutics for NSCLC is urgently needed.

The interactions between tumor cells and tumor microenvironment (TME) have a deep influence on cancer progression and contribute the mostly hallmarks of cancer (Hanahan and Weinberg, 2011). TME has been the new target of cancer treatment (Fang and Declerck, 2013), which is composed of many different non-cancerous cell types in addition to cancer cells, including endothelial cells, adipocytes, fibroblasts, myofibroblasts, pericytes, smooth muscle cells, and various immune cells (Quail and Joyce, 2017). Among these cells, immune cells play an important role in regulating anti-tumor immunity. Over the last decade, the influence of host immune cells has emerged as a critical determinant of cancer biology and a key factor in the success or failure of human cancer therapy (Emens et al., 2012).

Yu-Ping-Feng (YPF), as an ancient Chinese herbal decoction, is derived from the Dan-Xi Xin Fa by ZHU Dan-Xi of Chinese Yuan Dynasty and has been used for the treatment of cold and flu for several centuries in clinical practice. YPF is composed of three herbal medicines, i.e. Huang Qi (*Astragali Radix*), Bai Zhu (*Atractylodis Macrocephalae Rhizoma*), and Fang Feng (*Saposhnikoviae Radix*) in a weight ratio of 2:2:1. It has been used in the treatment of lung cancer (Chen, 2013; Chen et al., 2013) and the application of preventing viral infections like SARS, due to its immunomodulatory effects. It was shown to enhance cellular immunity too. Moreover, our previous studies also have demonstrated that YPF significantly inhibited the growth of Lewis lung cancer (LLC) cells which were inoculated subcutaneously, prolonged the survival of tumor-bearing mice (Luo et al., 2016). However, the regulation mechanism of YPF in the tumor microenvironment remains unknown.

In the present study, we investigated the therapeutic effects of YPF on orthotopic LLC tumor model and its regulations towards various immune cells in spleen and tumor. We found that YPF increased the percentages of CD4^+^ T cells and macrophages in tumor microenvironment, and the cytotoxicity of CD4^+^ T cells by inducing M1 macrophage polarization. Thus, YPF was a potent regulator of M1 macrophage polarization and might have a promising application in tumor immunotherapy.

## Materials and Methods

### Yu-Ping-Feng Preparation and Chemical Structure Analysis by HPLC

**Figure 1:**
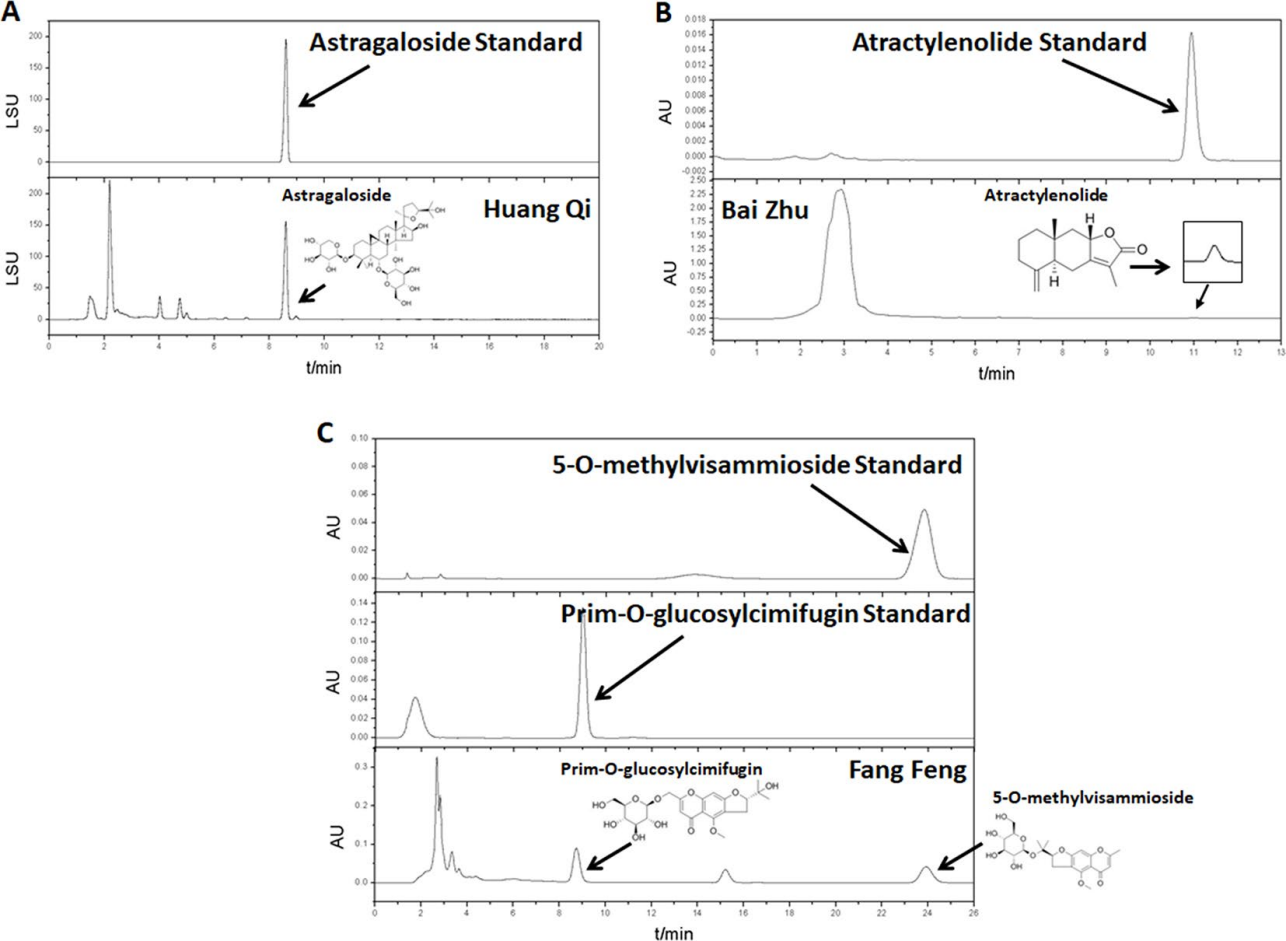
The water extracts of Huang Qi, Bai Zhu, and Fang Feng were analyzed by high performance liquid chromatography (HPLC). **(A)** Huang Qi, using astragaloside as the positive control. **(B)** Bai Zhu, using atractylenolide as the positive control. **(C)** Fang Feng, using prim-O-glucosylcimifugin and 5-O-methylvisammioside as the positive controls according to China Pharmacopoeia 2015 Edition.

Huang Qi (*Astragali Radix*, batch number: 150814), Bai Zhu (*Atractylodis Macrocephalae Rhizoma*, batch number: 150818), and Fang Feng (*Saposhnikoviae Radix*, batch number: 150805), as tablets, were purchased from Shanghai Kangqiao Chinese Medicine Tablet Co., LTD. (Shanghai, China). These tablets were processed separately but using the same method. First, the tablet was soaked with stilled water in a beaker for 2 h. Second, to obtain the first decoction, an addition of 10-fold water (1:10, w/v) was added to the beaker. The mixture was then boiled for 1.5 h and filtered. Third, for the second decoction, an addition of 8-fold water (1:8, w/v) was added to the beaker that consisted Chinese medicine tablet residue. The mixture was then boiled for 1h and filtered. Lastly, the two decoctions were mixed together and concentrated. After freeze-dried, they were stored in −80°C. As quality control, high performance liquid chromatography (HPLC) analysis of Huang Qi, Bai Zhu, and Fang Feng was performed on an Agilent 1260 liquid chromatography system (Agilent Technologies Inc., USA). All preparative reagents were filtered through a 0.22mm organic membrane. Astragaloside (TAUTO BIOTECH, CAS: 84687-43-4), atractylenolide (TAUTO BIOTECH, CAS: 73069-14-4), prim-O-glucosylcimifugin (TAUTO BIOTECH, CAS: 80681-45-4), and 5-O-methylvisammioside (TAUTO BIOTECH, CAS: 84272-85-5) were the positive controls according to China Pharmacopoeia 2015 Edition. Astragaloside (5mg), atractylenolide (40 µg), prim-O-glucosylcimifugin (60 µg) and 5-O-methylvisammioside (60 µg) were prepared with 1 ml methanol, then filter by Nylon membrane filter (0.22 µm). The solution was further analyzed by HPLC. The bioactive components identified were astragaloside (C_41_H_68_O_14_), atractylenolide (C_15_H_20_O_2_), prim-O-glucosylcimifugin (C_22_H_28_O_11_) and 5-O-methylvisammioside (C_22_H_28_O_10_). As shown in **Figure 1**, the bioactive components were extracted including astragaloside, atractylenolide, prim-O-glucosylcimifugin and 5-O-methylvisammioside in Huang Qi, Bai Zhu, and Fang Feng. The results were the same as our previous research (Luo et al., 2016). **Table 1** also showed the content of these dominating compounds in different drugs. To compose Yu-Ping-Feng (YPF), Huang Qi, Bai Zhu, and Fang Feng freeze-dried powders were mixed at the ratio of 2:2:1 (calculated basing on the crude drug dose). According to the results of **Table 1**, we could know the content ratio of each bioactive component in YPF. Then we prepared different concentration YPF to do further study. *In vivo*, 585 mg/ml of YPF was administered *via* intragastric, whereas 1 mg/ml, 0.5 mg/ml, 0.25 mg/ml, 0.125 mg/ml of YPF were used to treat cells *in vitro*.

**Table 1:** Content of dominating compounds.

Name	Chemical formula	Source	Content (%)
Astragaloside	C_41_H_68_O_14_	*Astragali Radix*	0.053
Atractylenolide	C_15_H_20_O_2_	*Atractylodis Macrocephalae Rhizoma*	0.00056
Prim-O-glucosylcimifugin	C_22_H_28_O_11_	*Saposhnikoviae Radix*	0.34
5-O-methylvisammioside	C_22_H_28_O_10_	*Saposhnikoviae Radix*	0.35

### Reagents and Antibodies

Reagents were purchased as follows: Hygromycin B (Roche, 31282-04-9), Matrigel Matrix (BD, 356234), D-luciferin (MCE, 2591-17-5), Collagenase I (YESEN, 40507ES60), Dnase I (YESEN, 10607ES15), True-Nuclear^™^ Transcription Factor Buffer Set (Biolegend, 424401), PrimeScript^™^ RT Master Mix (Takara, RR036A), SYBR Premix Ex Taq (Takara, RR420A), EasySep^™^ Mouse CD4^+^ T Cell Isolation Kit (STEMCELL Technologies, 19852). The antibodies used for flow cytometry were purchased as follows: PE anti-mouse CD3 (Biolegend, 100205), FITC anti-mouse CD4 (Biolegend, 100406), PE/Cy5 anti-mouse CD8 (Biolegend, 100710), APC anti-mouse NKp46 (Biolegend, 137608), Alexa Fluor ^®^ 647 anti-mouse Foxp3 (Biolegend, 126408), FITC anti-mouse CD11b (Biolegend, 101205), PE anti-mouse F4/80 (Biolegend, 123110), PE anti-mouse Gr-1 (Biolegend, 108407), APC anti-mouse CD107α (Biolegend, 121613), APC anti-mouse CD16/32 (Biolegend, 101325), APC anti-mouse CD206 (Biolegend, 141707), PE anti-mouse I-A/I-E (Biolegend, 107607). The primary antibodies used for Western blot were purchased as follows: iNOS (D6B6S) Rabbit mAb (Cell Signaling Technology, 13120), Arginase-1 (D4E3M^™^) Rabbit mAb (Cell Signaling Technology, 93668), STAT1 (D1K9Y) Rabbit mAb (Cell Signaling Technology, 14994), Phospho-Stat1 (Tyr701) (58D6) Rabbit mAb (Cell Signaling Technology, 9167), β-Actin (13E5) Rabbit mAb (Cell Signaling Technology, 4970). The second antibody used for Western blot was purchased as follow: horseradish peroxidase (HRP)-conjugated goat anti-rabbit IgG (Cell Signaling Technology, 7074).

### Animals and Cell Lines

This animal study was carried out in accordance with the principles of the Basel Declaration and recommendations of Laboratory animal-Guideline for ethical review of animal welfare (GB/T 35892-2018), Shanghai University of Traditional Chinese Medicine Institutional Animal Care and Use Committee. The protocol was approved by Shanghai University of Traditional Chinese Medicine Institutional Animal Care and Use Committee. All animals were euthanized in a CO_2_ chamber. The male C57BL/6 mice with the age of 5 weeks and body weight of 20 ± 2g were purchased from Shanghai SLAC Laboratory Animal Co., LTD., and maintained in a specific pathogen-free environment. Conditions were as follows: the light/dark cycle repeated every 12h; the temperature was maintained at 20–22 °C; and the relative humidity was maintained at 50 ± 10%. After a one-week acclimation, mice were divided into control and YPF groups (*n* = 7 for survival analysis and n = 4 for other animal experiments). The mice were subjected to the intragastric administration of YPF at the daily dose of 117 mg per mouse (equal to 45 g of clinical dose, according to the record in the Dan-Xi Xin Fa by ZHU Dan-Xi of Chinese Yuan Dynasty) or the same volume of normal saline as the control for 14 consecutive days before the tumor cells inoculation. Mice were sacrificed at Day 14 for all animal procedures expect survival study. Mouse primary peritoneal macrophages were prepared from female C57BL/6 mice (4-6 weeks of age) as described previously (Zhang et al., 2017). The purity of primary peritoneal macrophages was performed by Flow cytometric analysis. Mouse CD4^+^ T cells were separated from C57BL/6 mice spleen with EasySep^™^ Mouse CD4^+^ T Cell Isolation Kit (Stem Cell Technologies, Canada). Lewis lung cancer Luciferase (LLC-Luc) cells, which were transfected with Luciferase plasmid, were conserved in our own laboratory. The cells were maintained in DMEM medium (Hyclone, USA) supplemented with 10% fetal bovine serum (FBS), 10% penicillin (100 U/ml), streptomycin (100 U/ml) (Invitrogen Corporation, USA), and 250 µg/ml Hygromycin B (Roche, Switzerland). The RAW264.7 murine macrophage cells were obtained from Shanghai Cell Bank of Chinese Academy of Sciences. The cells were maintained as described above expect Hygromycin B. Cells were cultured in a humid incubator with 5% CO_2_ at 37°C.

### Orthotopic Lung Tumor Implantation and Survival Study

Mice were anesthetized using 10mg/kg of pentobarbital sodium *via* intraperitoneal injection before inoculating the orthotopic lung tumor. A 1–1.5cm incision was made on left chest side, about 1cm under the left axillary front. Muscles and fat were separated to visualize the lung movement. LLC-Luc cells suspended in 100 µl non-serum DMEM/matrigel were injected directly into left lung tissues at the depth of 2–3 mm. Then stitched the wound and sprayed some gentamycin and erythromycin on the incision. Mice were allowed to recover in a preheated incubator for 30 min. Mice were sacrificed when Body Condition Scoring was 2 or less, or at 20% weight loss.

### Mice Bioluminescence Imaging

Mice bioluminescence imaging was performed once a week after the tumor cells inoculation to monitor orthotopic lung tumor growth. Mice were injected with D-luciferin *via* intraperitoneal at 150 mg/kg, anesthetized with 2% isoflurane and then imaged through Caliper IVIS Lumina XR Imaging System 15 min after D-luciferin injection. The Region of Interest (ROI) was defined as 3.2 cm radius circle over left lung area. Average radiance (p/s/cm^2^/sr) within ROI was quantified using Living Image.

### Mononuclear Cell Preparation

Mononuclear cells were isolated from lung tumor tissues by digesting the tissues with 1 mg/ml Collagenase I (YESEN, China) and 10 µg/ml Dnase I (YEASEN, China) at 37°C for 1 h. Mononuclear cells were isolated from spleen by grinding the tissues directly. These cells were pushed through 200 mesh screen and then treated with erythrocytolysin. Using PBS washed the mononuclear cells twice.

### Flow Cytometric Analysis

For extracellular flow cytometry, cells were exposed to appropriate antibodies for 30 min at 4°C, washed, and resuspended in PBS containing 1% of FBS. For intracellular flow cytometry, cell surface staining was performed as described above, and then treated with True-Nuclear^™^ Transcription Factor Staining (BioLegend, USA). Cells were exposed to appropriate amount of fluorochrome conjugated antibody for detection of intracellular antigen and incubated in the dark at room temperature for 30 min, washed, and resuspended in PBS containing 1% of FBS. Data were acquired using BD Accuri C6 (BD Biosciences) instrument and analyzed using the FlowJo software (Ashland, OR).

### Osmotic Pressure Assay

To access the influence of YPF on osmotic pressure of medium, YPF freeze-dried powder was dissolved to 128 mg/ml by using DMEM medium, and then diluted to different concentrations (64, 32, 16, 8, 4, 2, 1, 0.5, 0.25, and 0.125 mg/ml). The osmotic pressure was acquired by freezing point osmotic pressure meter (Gonotec, Germany).

### Cell Viability Assay

To evaluate the influence of YPF on cell viability, cells were seeded into 96-well plates at a density of 5 × 10^3^ (LLC cells and RAW264.7 cells) per well, and treated with 0.125–2 mg/ml of YPF for 24 or 48 h. The cell viability was measured by MTT following manufacturer’s instructions. The absorbance was read at 490 nm using Synergy 2 Muti-Mode Microplate Reader (BioTek Instrument, Int., VT). Three independent experiments were performed.

### Biophotonic Cell Killing Activity Assay

To assess the killing activity of macrophages and CD4 ^+^ T cells, biophotonic cell killing activity assay was performed as described previously (Gong et al., 2017). Briefly, mouse primary peritoneal macrophages and mouse CD4^+^ T cells together or alone co-incubated with LLC-Luc cells at a 1:1:1 ratio with or without different concentrations of YPF at 37°C. After 24h, luciferin (Invitrogen, USA) was added to a final concentration of 0.14 mg/ml into each well. Luminescence flux was read at 590/35nm using Synergy 2 Multi-Mode Microplate Reader (BioTek Instrument, Int., Winooski, VT). Percent viability was calculated as the mean luminescence of the test sample (MEAN_TEST_) minus background (MEAN_SDS_) divided by the mean luminescence of the input number of target cells used in the assay (MEAN_media_) minus background (MEAN_SDS_). The percentage lysis is calculated according to the formula [1−(MEAN_TEST_−MEAN_SDS_)/(MEAN_media_−MEAN_SDS_)] × 100%.

### Degranulation Assay

To assess the killing activity of T cells, degranulation assay was used. Mononuclear cells were isolated from lung tumor tissues as described above, and then cultured alone or with LLC-Luc cells at 1:1 ratio for 4 h at 37°C. Mouse primary peritoneal macrophages and mouse CD4^+^ T cells together co-incubated with LLC-Luc cells at a 1:1:1 ratio with or without different concentrations of YPF at 37°C. After 24h, CD4^+^ T cells were separated from the mixed cells, and cultured alone or with LLC-Luc cells at 1:1 ratio for 4 h at 37°C. Meanwhile, for each assay, anti-mouse CD107α antibody or isotype IgG (BioLegend, USA) was added and incubated for 4h, and then T cells were stained with anti-mouse CD3 antibody, anti-mouse CD4 antibody, and anti-mouse CD8 antibody (BioLegend, USA) for 30 min at 4°C. CD107α expression on the surface of T cells was acquired by BD Accuri C6 (BD Biosciences) instrument. Data was analyzed using the FlowJo software (Ashland, OR).

### Quantitative Real-Time PCR

Lung tumor tissues were separated from mice which were sacrificed at Day 14 after orthotopic lung tumor implantation. RAW264.7 cells were seeded into 6-well plates at a density of 5×10^5^ per well, and treated with different concentrations of YPF for 24h. Total RNA was extracted from lung tumor tissues and RAW264.7 cells by using TRIzol reagent (Thermo Fisher, USA) according to the manufacturer’s protocols. Reverse transcription was performed with the PrimeScript^™^ RT Master Mix kit (TaKaRa, Japan), and QPCR was performed with the SYBR Premix Ex Taq (TaKaRa, Japan) on ABI system (Applied Biosystems, Life Technologies). The PCR protocol included one cycle at 95°C (30 s) followed by 40 cycles of 95°C (5 s) and 60°C (30 s). The expression of β-actin mRNA was used as a standard. **Table 2** showed all the primer sequences in the experiments.

**Table 2:** Primers used for real-time PCR analysis.

Genes		Primer sequence
β-actin	Forward primer	5′-CCACCATGTACCCAGGCATT-3′
	Reverse primer	5′-AGGGTGTAAAACGCAGCTCA-3′
TNF-α	Forward primer	5′-GCTCTTCTGTCTACTGAACTTCGG-3′
	Reverse primer	5′-ATGATCTGAGTGTGAGGGTCTGG-3′
IFN-γ	Forward primer	5′-AGTGGCATAGATGTGGAAGAAAAGA-3′
	Reverse primer	5′-TCAGGTGTGATTCAATGACGCTTAT-3′
TGF-β	Forward primer	5′-ATCTCGATTTTTACCCTGGTGGT-3′
	Reverse primer	5′-CTCCCAAGGAAAGGTAGGTGATAGT-3′
IL-1β	Forward primer	5′-AGTTGACGGACCCCAAAAG-3′
	Reverse primer	5′-TTTGAAGCTGGATGCTCTCAT-3′
IL-2	Forward primer	5′-CTGAGCAGGATGGAGAATTACA-3′
	Reverse primer	5′-AGGTCCAAGTTCATCTTCTAGGC-3′
IL-4	Forward primer	5′-TTGTCATCCTGCTCTTCTTTCTCG-3′
	Reverse primer	5′-CTCACTCTCTGTGGTGTTCTTCGTT-3′
IL-10	Forward primer	5′-AAACAACTCCTTGGAAAACCTCG-3′
	Reverse primer	5′-TCCCCAATGGAAACAGCTTAAAC-3′
IL-12	Forward primer	5′-AGTGACATGTGGAATGGCGT-3′
	Reverse primer	5′-CAGTTCAATGGGCAGGGTCT-3′
iNOS	Forward primer	5′-CTGCAGCACTTGGATCAGGAACCTG-3′
	Reverse primer	5′-GGGAGTAGCCTGTGTGCACCTGGAA-3′
Arg-1	Forward primer	5′-CAGAAGAATGGAAGAGTCAG-3′
	Reverse primer	5′-CAGATATGCAGGGAGTCACC-3′

### Western Blot

RAW264.7 cells were seeded into 6-well plates at a density of 5 × 10^5^ per well, and treated with different concentrations of YPF for 24 h. Then the cells were lysed with RIPA lysis buffer, and quantitated using BCA protein reagent assay kit (YEASEN, China) and analyzed by 8% of SDS-PAGE, followed by immunoblotting using enhanced chemiluminescence substrate (Merck Millipore) according to the manufacturer’s instructions. Bands were visualized using a chemiluminescent detection system (Bio-Rad). The expression of β-actin protein was used as a standard.

### Statistical Analysis

Data were expressed as means ± standard deviation (SD), and *P* < 0.05 was considered statistically significant. The survival was determined using log-rank test by SPSS 18.0. Other statistical analyzes were performed using the Student’s t-test and one-way analysis of variance (ANOVA).

## Results

### YPF Prolongs Orthotopic Lung Tumor-Bearing Mouse Survival

**Figure 2:**
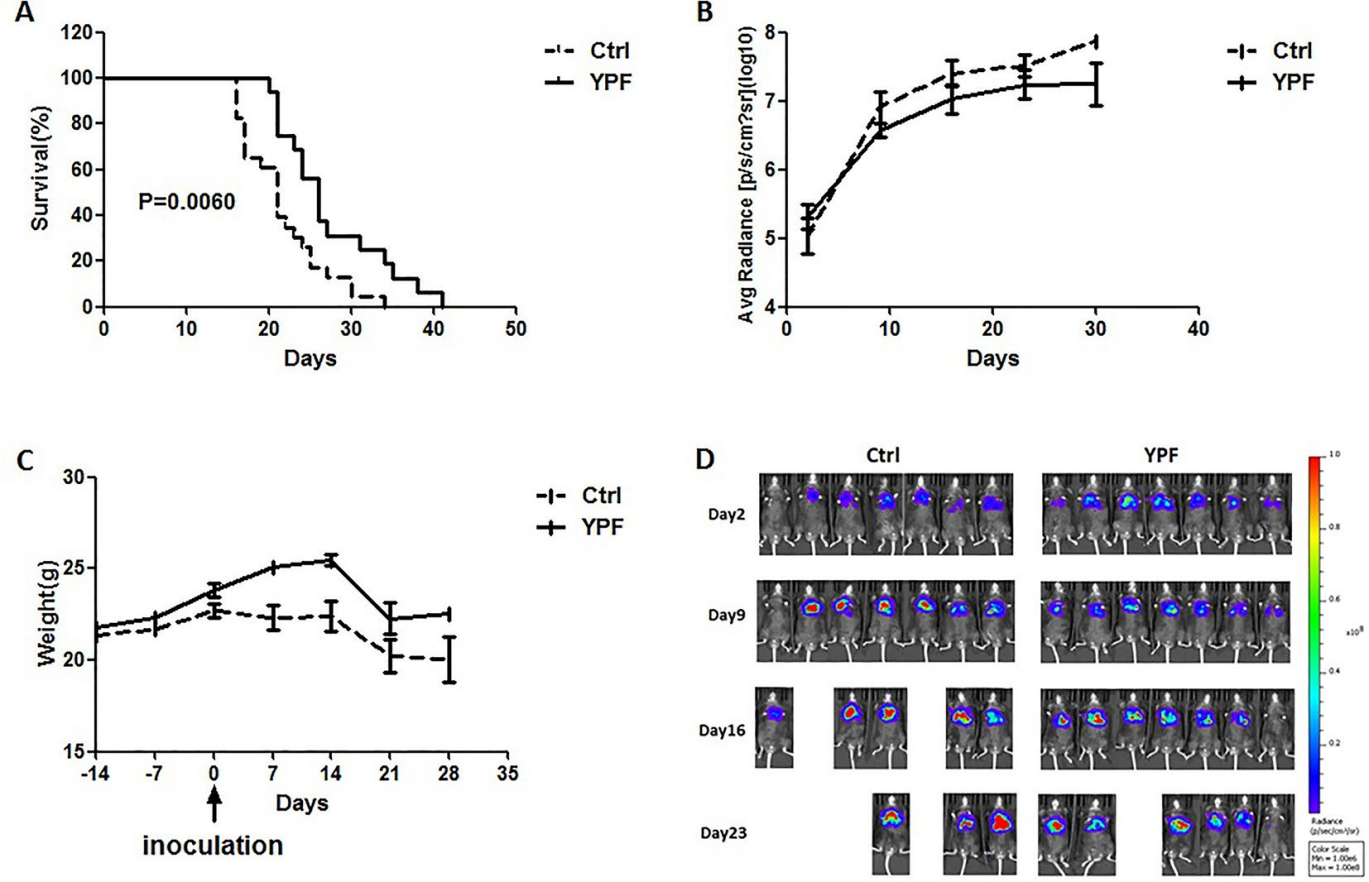
YPF prolonged the survival rate of orthotopic lung tumor-bearing mice, inhibited the growth of LLC tumor cells and reduced the decline of body weight. The LLC-Luc cells (5×10^5^ cells/mouse) were orthotopic inoculated in the left lung of C57BL/6 mice. The mice were administered with normal saline or YPF at a daily dose of 117mg for each mouse for 14 consecutive days before inoculation. **(A)** Survival curves, *P* = 0.0060. **(B)** The average radiance of tumor at different time points. **(C)** The weight of mice at different time points. **(D)** The photos of mice bioluminescence imaging at different time points.

To access the effect of YPF on NSCLC, an orthotopic luciferase stably expressed LLC (LLC-Luc) tumor model was established. YPF was administered at the daily dose of 117 mg for each mouse by gavage. Results showed that YPF significantly prolonged survival of orthotopic lung tumor-bearing mice when compared to the untreated control (*P* < 0.05) (**Figure 2A**). YPF inhibited LLC cell tumor growth according to the results of mice bioluminescence imaging, and reduced the decline of body weight, though there was no statistically significant (**Figures 2B–D**). Taken together, these results demonstrated that YPF could prolong the survival of orthotopic lung tumor-bearing mice and inhibited LLC cells growth *in vivo*.

### YPF Increases CD4^+^ T Cell and Macrophage Population in Tumor Microenvironment, and Induces the Th1 Immunity Response

**Figure 3:**
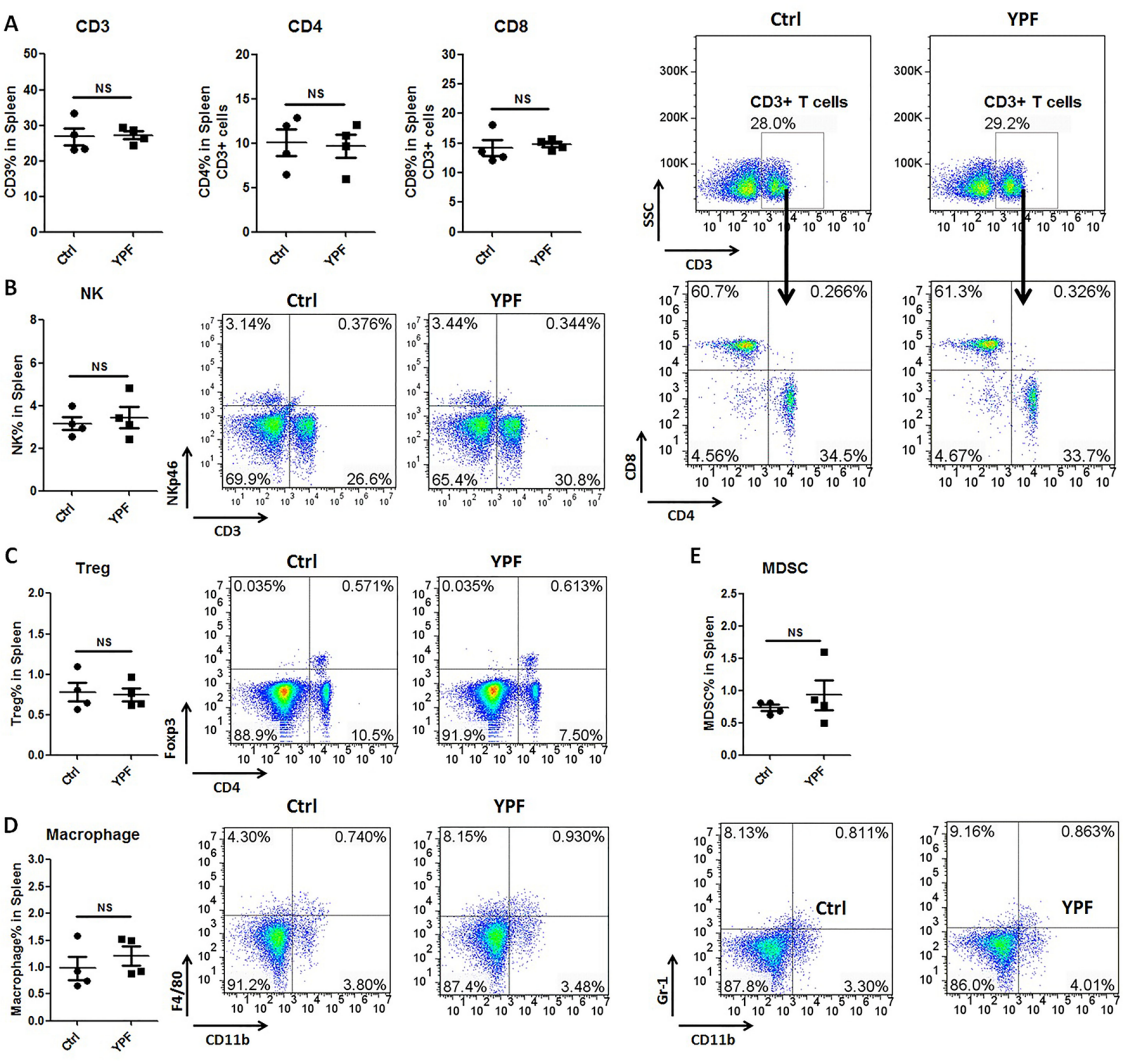
YPF did not influence the population of immunity cells in spleen. Mononuclear cells were isolated from the spleen of orthotopic LLC-bearing C57BL/6 mice treated with normal saline or YPF at Day 14 after inoculation and then analyzed by flow cytometry after stained with CD3, CD4, CD8, NKp46, Foxp3, CD11b, F4/80, and Gr-1. **(A)** Percentages of CD3^+^ T, CD4^+^ T, and CD8^+^ T cells in spleen. **(B)** Percentage of NK cells in spleen. **(C)** Percentage of regulatory T cells (Treg) in spleen. **(D)** Percentage of macrophage in spleen. **(E)** Percentage of myeloid-derived suppressor cells (MDSCs) in spleen. NS, non-significant.

**Figure 4:**
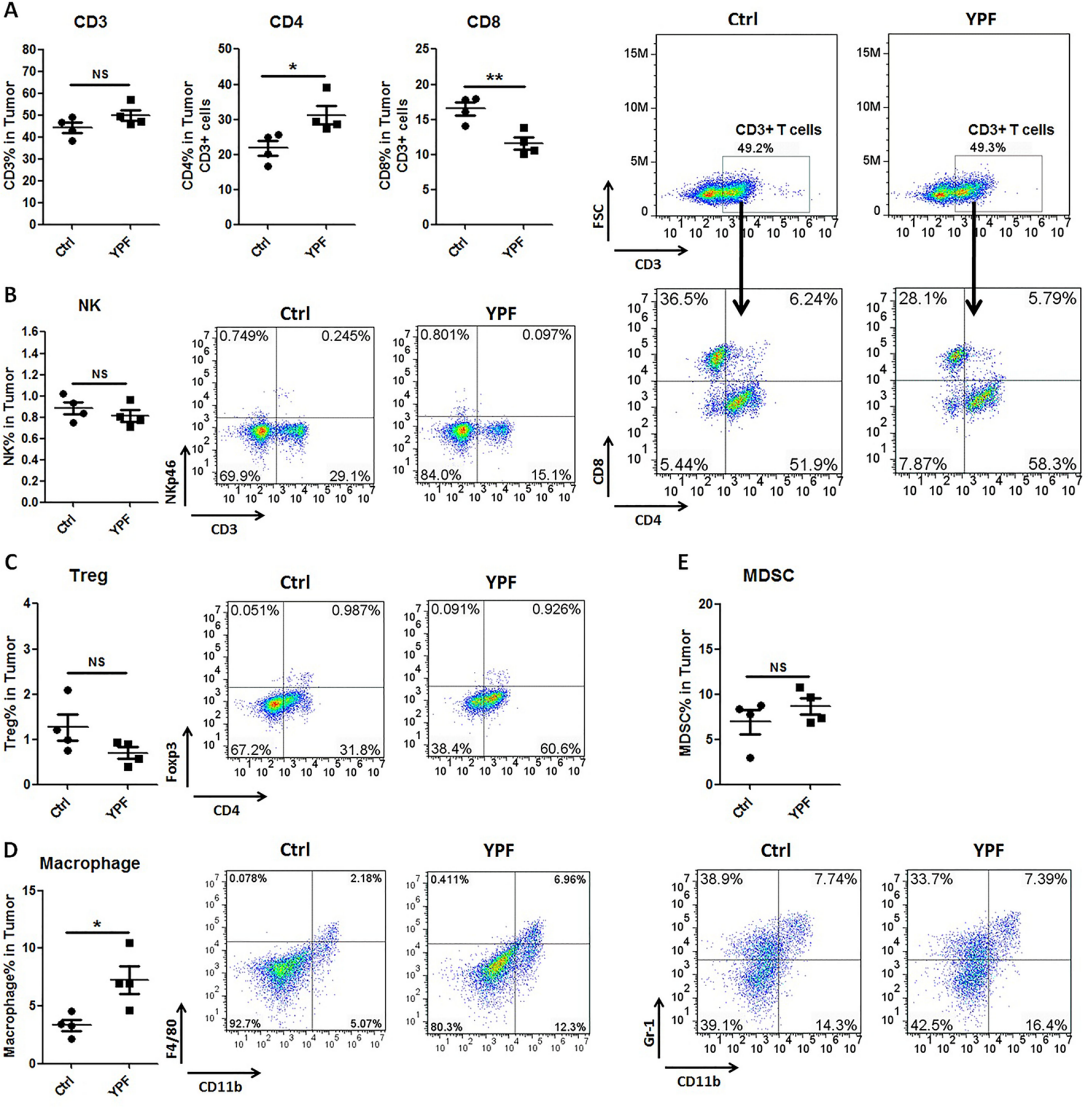
YPF significantly increased the population of CD4^+^ T cells and macrophages, and decreased the population of CD8^+^ T cells in tumor tissues. Mononuclear cells were isolated from tumor tissues of orthotopic LLC-bearing C57BL/6 mice treated with normal saline or YPF at Day 14 after inoculation and analyzed by flow cytometry after stained with CD3, CD4, CD8, NKp46, Foxp3, CD11b, F4/80, and Gr-1. **(A)** Percentages of CD3^+^ T, CD4^+^ T, and CD8^+^ T cells in tumors. **(B)** Percentage of NK cells in tumors. **(C)** Percentage of regulatory T cells (Treg) in tumors. **(D)** Percentage of macrophage in tumors. **(E)** Percentage of myeloid-derived suppressor cells (MDSCs) in tumors. *P < 0.05, **P < 0.01. NS, non-significant.

**Figure 5:**
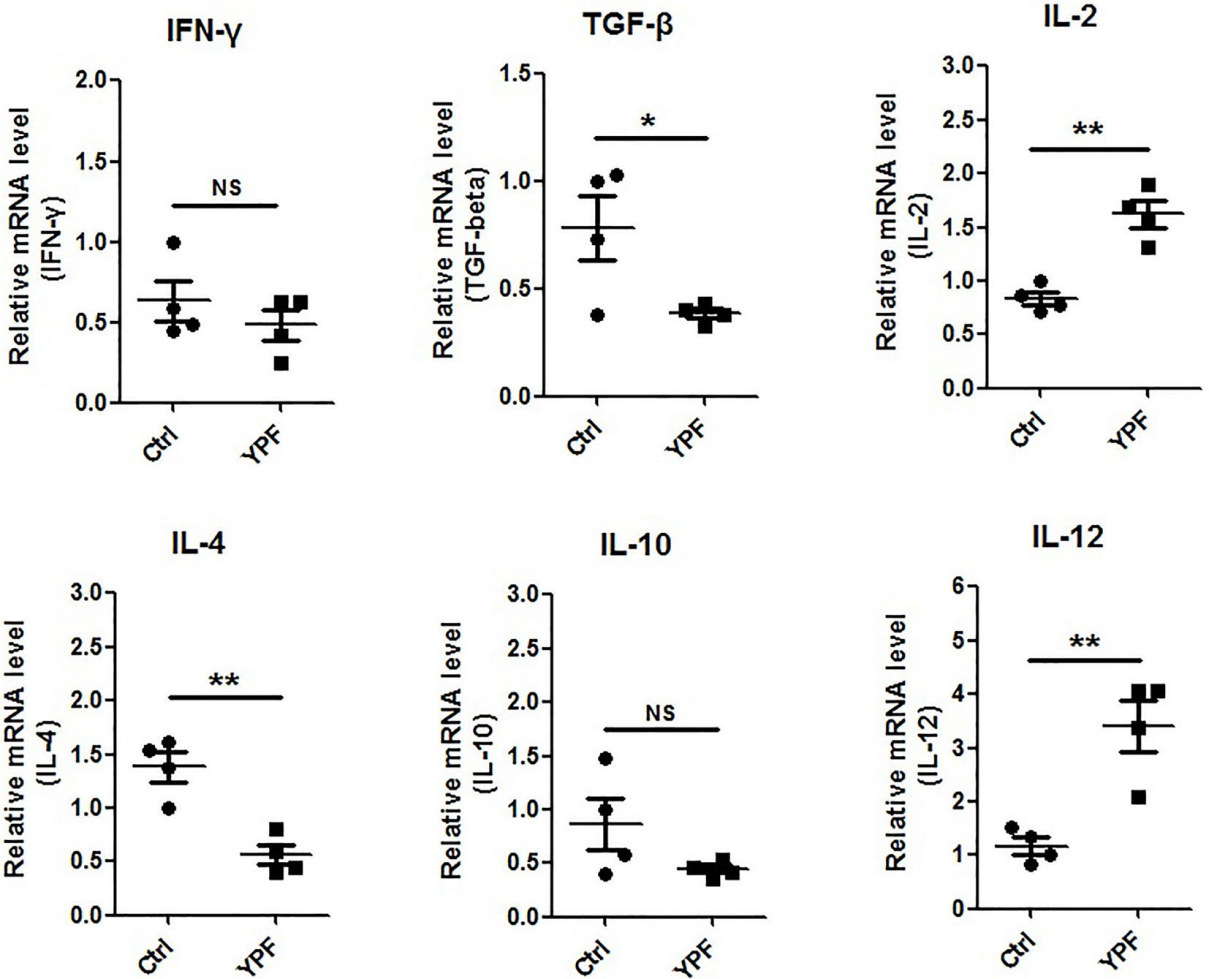
YPF increased the mRNA expression of IL-2, IL-12 and decreased the mRNA expression of TGF-β, IL-4 in tumor tissues. Orthotopic LLC-bearing mice were treated with normal saline or YPF as described previously. At Day 14 after inoculation, total RNA was isolated from tumor tissues and the mRNA expressions of IFN-γ, TGF-β, IL-2, IL-4, IL-10, and IL-12 were analyzed by real-time PCR. *P < 0.05, **P < 0.01. NS, non-significant.

To address the immune microenvironment, we analyzed the population of CD4^+^ T cells (CD3^+^CD4^+^), CD8^+^ T cells (CD3^+^CD8^+^), Natural killer cells (NK, CD3^−^NKp46^+^), regulatory T cells (Treg, CD4^+^Foxp3^+^), macrophages (CD11b^+^F4/80^+^), and myeloid-derived suppressor cells (MDSC, CD11b^+^Gr-1^+^) in spleen and tumor tissues by flow cytometry. In spleen, no significant effects were observed on the percentage of these cells (*P* > 0.05) (**Figure 3**). In tumor tissues, YPF significantly increased CD4^+^ T cells and macrophages population, and decreased CD8^+^ T cells population, but had no effect on other cells (**Figure 4**). Meanwhile, we analyzed the different cytokines expression in tumor tissues. Results showed that YPF increased the expression of IL-2 and IL-12, which were associated with Th1 immunity response, and decreased the expression of TGF-β and IL-4, which were related with Th2 immunity response (**Figure 5**). Taken together, these results suggested that the effect of YPF inhibited tumor growth might depend on CD4^+^ T cells and macrophages in tumor microenvironment.

### YPF Increases M1 Macrophages Population and Promotes the Cytotoxicity of CD4^+^ T Cells

**Figure 6:**
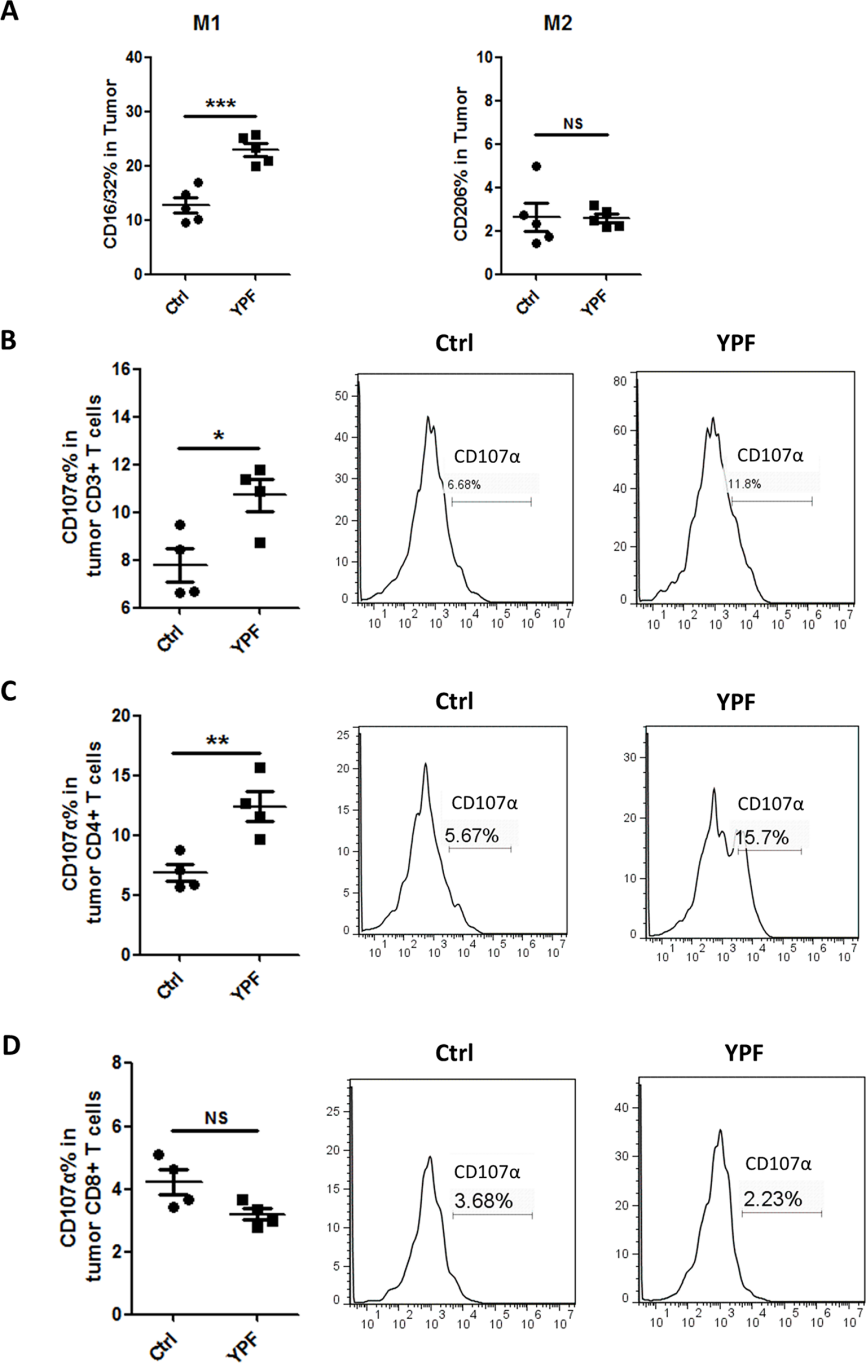
YPF significantly increased M1 macrophages population and promoted the cytotoxicity of CD4^+^ T cells in tumor tissues. Mononuclear cells were isolated from tumor tissues of orthotopic LLC-bearing C57BL/6 mice treated with normal saline or YPF at Day 14 after inoculation and analyzed by flow cytometry after stained with CD11b, F4/80, CD16/32, CD206, CD3, CD4, CD8, and CD107α. **(A)** Percentages of M1 and M2 macrophages in tumors. **(B)** The CD107α surface expression on CD3^+^ T cells in tumors. **(C)** The CD107α surface expression on CD4^+^ T cells in tumors. **(D)** The CD107α surface expression on CD8^+^ T cells in tumors. *P < 0.05, **P < 0.01, ***P < 0.001. NS, non-significant.

Since YPF increased percentages of macrophages and CD4^+^ T cells, we next determined the macrophage subsets and CD4^+^ T cell cytotoxicity. To address these issues, the populations of M1 (CD11b^+^F4/80^+^CD16/32^+^) and M2 macrophages (CD11b^+^F4/80^+^CD206^+^) and T cell degranulation were analyzed by flow cytometry. To evaluate T cell degranulation, the expression of CD107α was measured. CD107α appears on the cell surface following the fusion of lysosomes with the plasma membrane and is therefore used as a functional marker of degranulation of CD4^+^ T cells (Naito et al., 2015) and CD8^+^ T cells (Betts et al., 2003). Results showed that YPF did not induce an obvious change of M2 macrophage but significantly increased M1 macrophage population (**Figure 6A**). YPF clearly upregulated the CD107α surface expression on CD3^+^ T cells and CD4^+^ T cells, but slightly downregulated on CD8^+^ T cells (**Figures 6B–D**). Taken together, these results showed that YPF increased M1 macrophage and enhanced degranulation of CD4^+^ T cells.

### YPF Enhances CD4^+^ T Cell Cytotoxicity by Promoting the Macrophage Antigen Presentation

**Figure 7:**
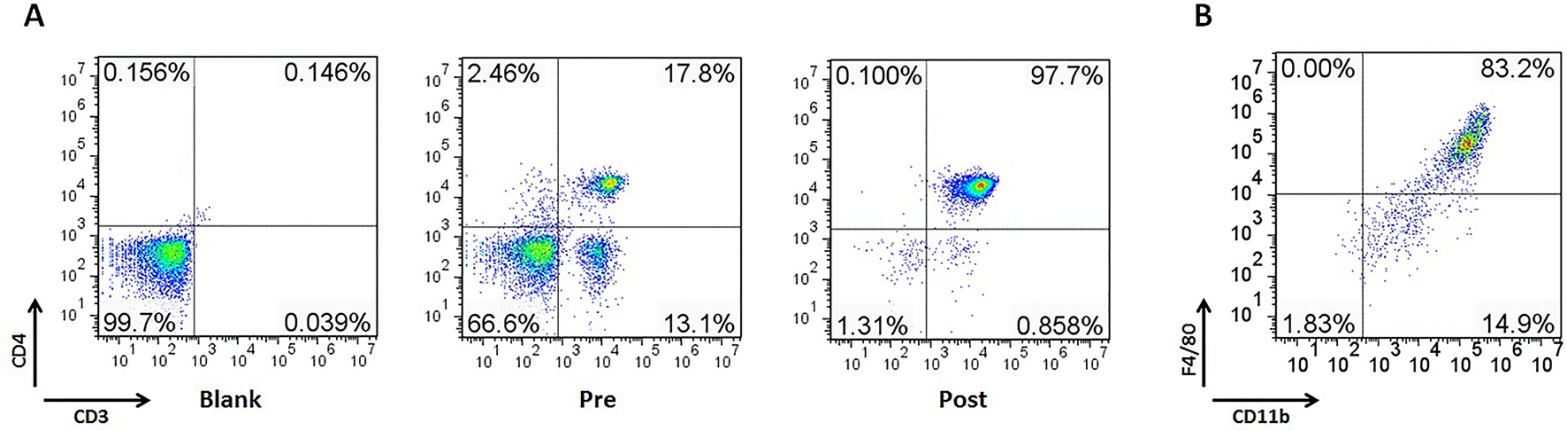
The purity of CD4^+^ T cells and primary peritoneal macrophages was up to 97.7% and 83.2%, respectively. Mouse CD4^+^ T cells were separated from C57BL/6 mice spleen with EasySep™ Mouse CD4^+^ T Cell Isolation Kit. Mouse primary peritoneal macrophages were prepared from female C57BL/6 mice (4–6 weeks of age) as described previously. **(A)** The purity of CD4^+^ T cells in spleen. **(B)** The purity of primary peritoneal macrophages.

**Figure 8:**
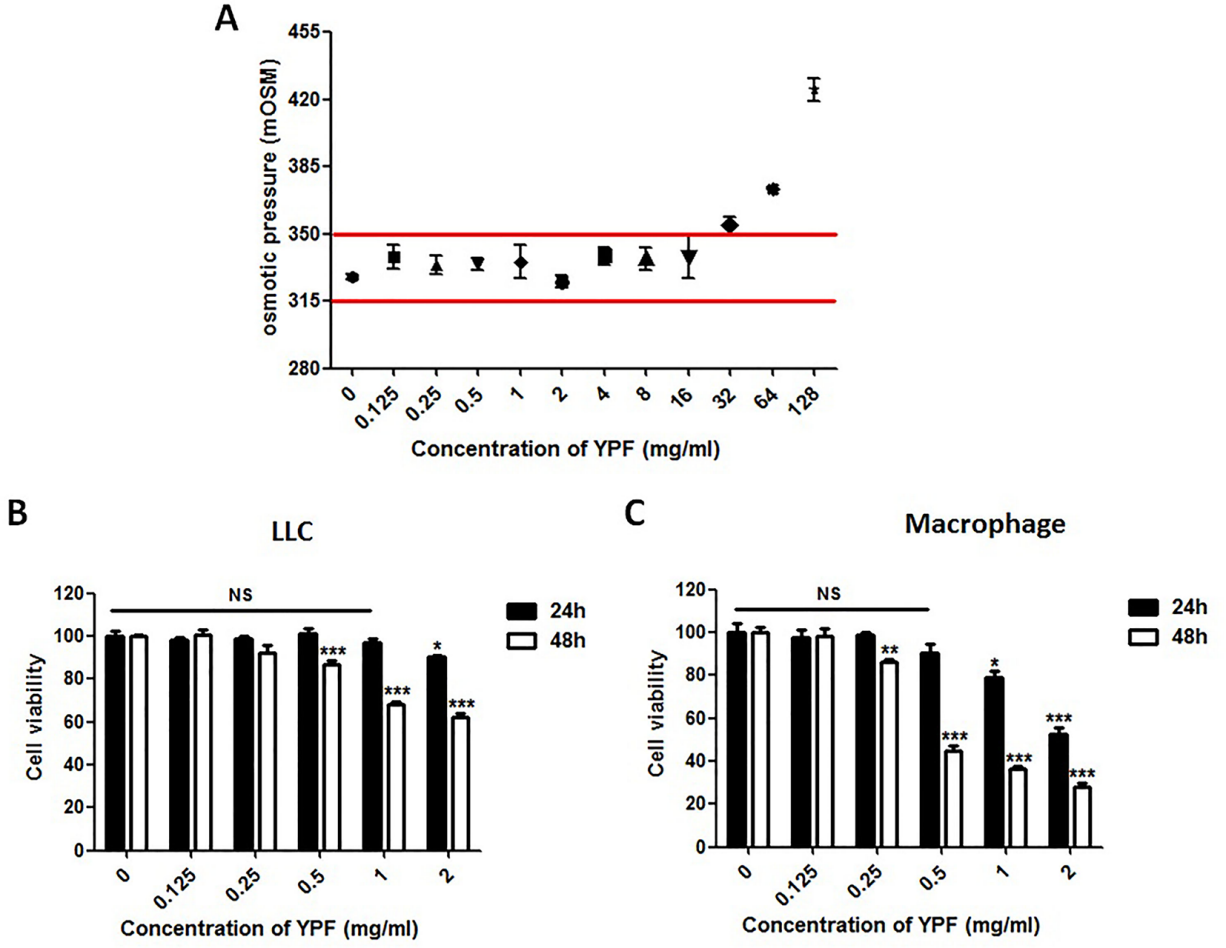
YPF within 1mg/ml did not affect the osmotic pressure of DMEM medium and the viability of LLC cells and macrophages in 24h. **(A)** The osmotic pressure of different concentrations YPF. **(B** and **C)** Effect of YPF on the viability of LLC and macrophages. Data were shown with means ± SD of at least three independent experiments. *P < 0.05, **P < 0.01, ***P < 0.001. NS, non-significant.

**Figure 9:**
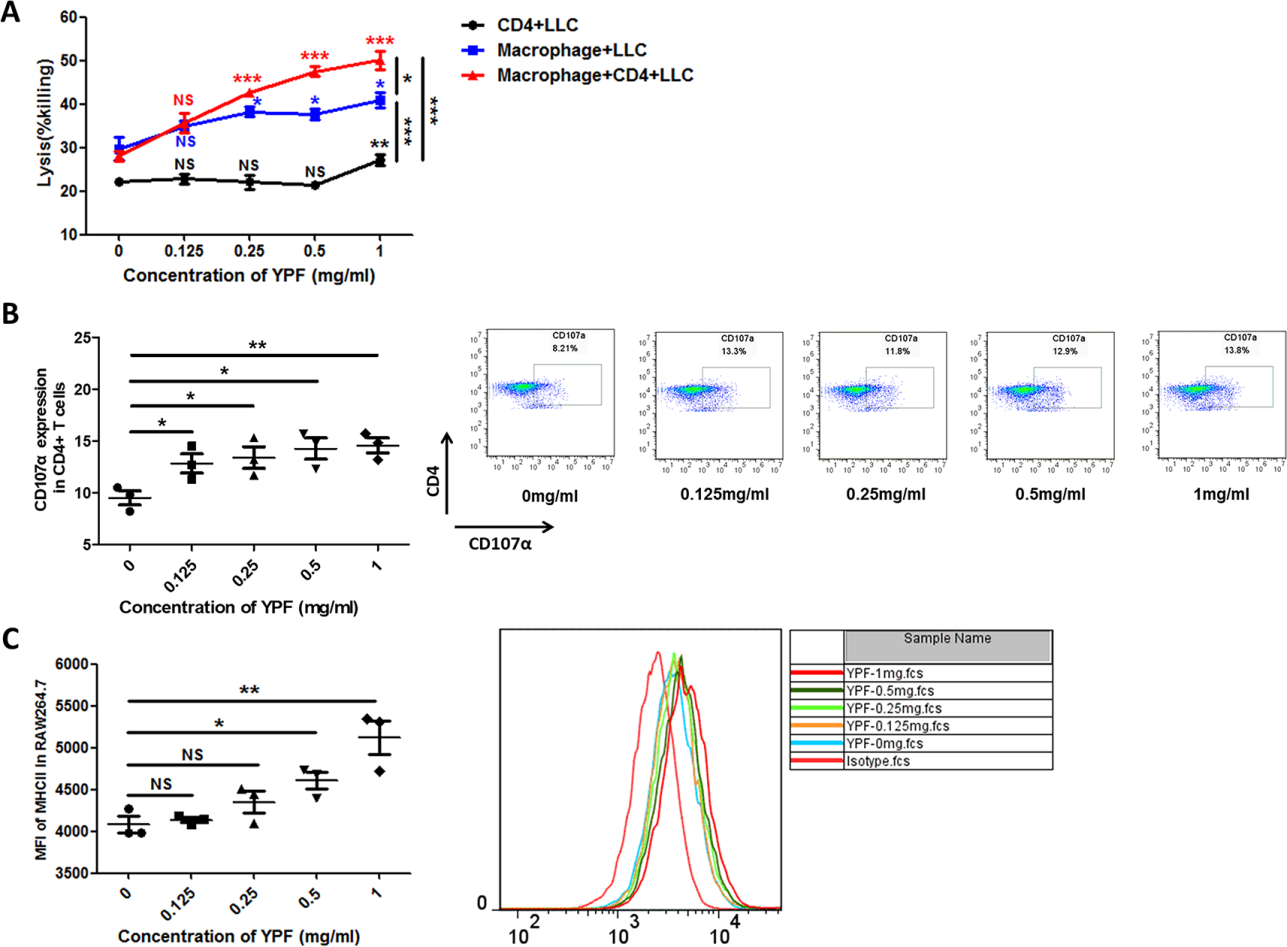
YPF significantly enhanced macrophage-mediated lysis of LLC in a concentration-dependent manner, and had no effect on CD4^+^ T cell-mediated lysis of LLC, but significantly increased CD4^+^ T cell-mediated lysis after co-incubated with macrophages. In addition, the enhancement of CD4^+^ T cell cytotoxicity by YPF was dependent on macrophage antigen presentation. **(A)** CD4^+^ T cell with or without macrophage mediated lysis of LLC-Luc cells. Mouse primary peritoneal macrophages and mouse CD4^+^ T cells together or alone co-incubated with LLC-Luc cells at a 1:1:1 ratio with or without different concentrations of YPF at 37°C for 24h. The specific lysis was evaluated by biophotonic luciferase assay as described in Methods. **(B)** The CD107α surface expression on CD4^+^ T cells was assessed by flow cytometry. **(C)** The I-A/I-E (MHC II) expression of macrophages. Macrophages were incubated with different concentrations of YPF for 24h. I-A/I-E expression on the surface of macrophages was analyzed by flow cytometry. Data were shown with means ± SD of at least three independent experiments. *P < 0.05, **P < 0.01, ***P < 0.001. NS, non-significant.

The above results showed that YPF increased M1 macrophage and enhanced degranulation of CD4^+^ T cells. M1 macrophage is a classical antigen presenting cell, this led us to determine whether the enhancement of CD4^+^ T cell degranulation is dependent on macrophages. To illuminate this issue, LLC-Luc cells, purified CD4^+^ T cells, and primary peritoneal macrophages were co-cultured. The purity of CD4^+^ T cells and primary peritoneal macrophages was detected by flow cytometry. As shown in **Figure 7**, the purity of CD4^+^ T cells and primary peritoneal macrophages was up to 97.7% and 83.2%, respectively. To optimize the concentration of YPF *in vitro*, the osmotic pressure assay and cell viability assay were performed to evaluate potential drug-induced toxicity. As shown in **Figure 8A**, YPF within 1 mg/ml did not affect the osmotic pressure of DMEM medium, and also did not influence the viability of LLC cells and macrophages in 24 h (**Figures 8B, C**). Therefore, YPF within 1 mg/ml was used for the subsequent studies *in vitro*. To analyze CD4^+^ T cell or macrophage mediated lysis of tumor cells, the biophotonic cytotoxicity assay was performed as described in Materials and Methods. Results showed that YPF significantly enhanced macrophage-mediated lysis of LLC in a concentration-dependent manner, and had no effect on CD4^+^ T cell-mediated lysis of LLC, but significantly increased CD4^+^ T cell-mediated lysis after co-incubated with macrophages (**Figure 9A**). To further access the effect of YPF on the cytotoxicity of CD4^+^ T cells in the co-culture system, CD107α surface expression on CD4^+^ T cells was detected by flow cytometry. Results showed that YPF clearly upregulated the CD107α surface expression on CD4^+^ T cells in a concentration-dependent manner (**Figure 9B**). These results suggested that YPF enhanced CD4^+^ T cell cytotoxicity might be dependent on macrophages. Additionally, CD4^+^ T cells activation depends on the present antigens by professional antigen-presenting cells (APCs), such as macrophages and dendritic cells, in a major histocompatibility complex (MHC) class II-dependent manner. This led us to investigate whether YPF had an effect on the I-A/I-E (MHC II) expression on macrophages. Results showed that YPF significantly promoted the I-A/I-E expression on macrophages in a concentration-dependent manner (**Figure 9C**). Taken together, these observations indicated that the enhancement of CD4^+^ T cell cytotoxicity by YPF was dependent on macrophage antigen presentation.

### YPF Induces M1 Macrophage Polarization

**Figure 10:**
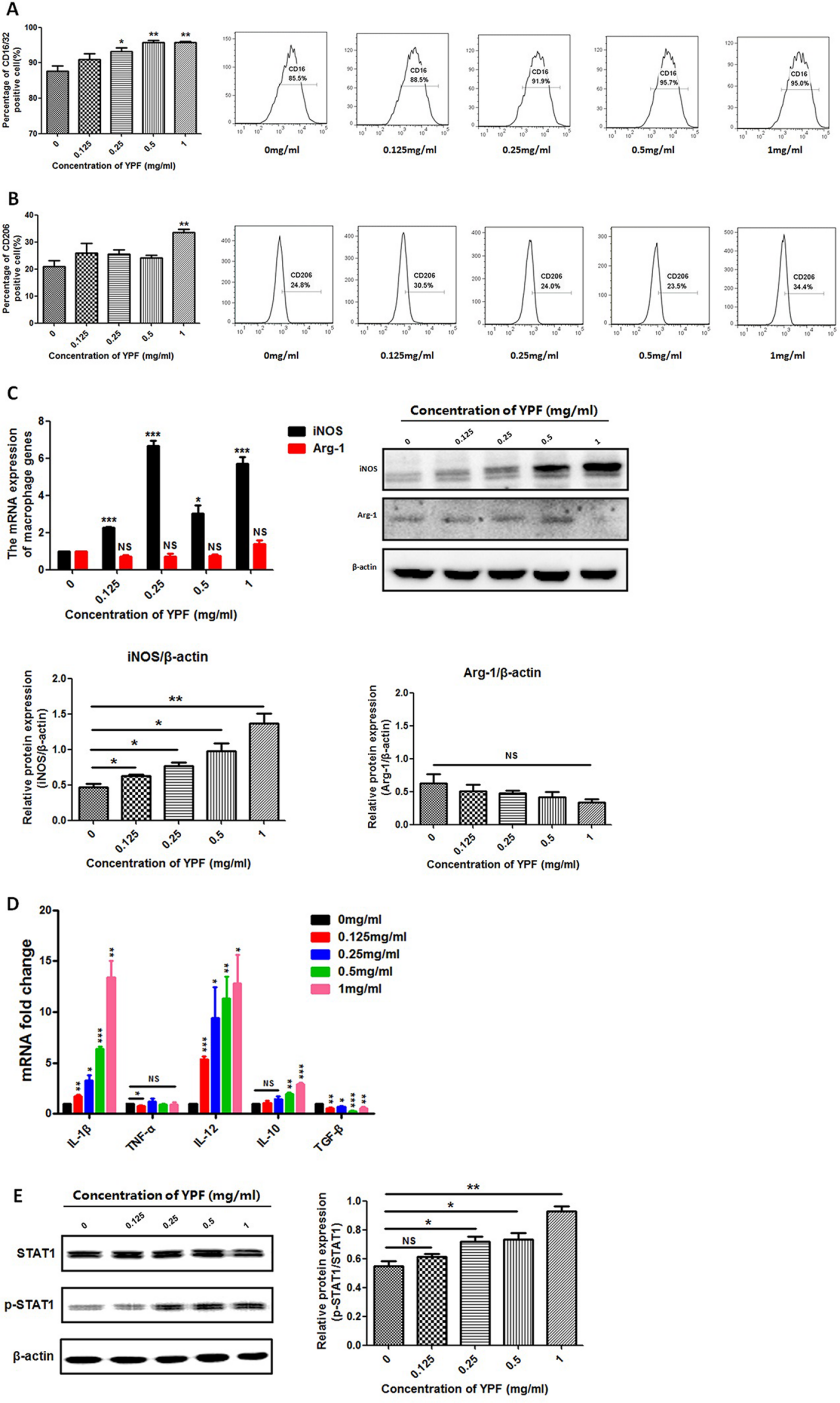
YPF induced M1 macrophage polarization through promoting STAT1 activation. Macrophages were incubated with different concentrations of YPF for 24h. The CD16/32 or CD206 expression of macrophages was analyzed by flow cytometry. The expressions of iNOS and Arg-1 were evaluated by real-time PCR and Western Blot. Total RNA was isolated from macrophages and the mRNA expressions of IL-1β, TNF-α, IL-12, IL-10 and TGF-β were analyzed by real-time PCR. Protein was isolated from macrophages and the protein expressions of STAT1, p-STAT1 were analyzed by Western Blot. **(A)** The CD16/32 expression of macrophages. **(B)** The CD206 expression of macrophages. **(C)** The mRNA and protein expressions of iNOS, Arg-1 in macrophages. **(D)** The mRNA expressions of different cytokines in macrophages. **(E)** The protein expressions of STAT1 and p-STAT1 in macrophages. Data were shown with means ± SD of at least three independent experiments. *P < 0.05, **P < 0.01, ***P < 0.001. NS, non-significant.

The macrophage function as antigen presentation is associated with the distinct macrophage subsets. Since YPF promoted the antigen presentation of macrophage, we next explored whether YPF induced M1 macrophage polarization. The phenotypes of the RAW264.7 cells were determined by measuring the surface markers CD16/32 (M1) and CD206 (M2). Compared to control group (0 mg/ml), a significant increase was observed in CD16/32 positive cells but not in CD206 positive cells when the RAW264.7 cells were cultured with different concentrations of YPF (**Figures 10A, B**). Furthermore, iNOS and Arg-1 are usually used to distinguish M1 and M2 macrophages. Therefore, the expressions of iNOS and Arg-1 were detected by real-time PCR and western blot. Results showed that YPF increased iNOS mRNA and protein expressions in RAW264.7 cells, but had no effect on Arg-1 expression (**Figure 10C**). We next analyzed IL-1β, TNF-α, IL-12, IL-10, TGF-β mRNA expressions in RAW264.7 cells by real-time PCR. Results showed that YPF could increase expression of IL-1β, IL-12, which were associated with M1 polarization (**Figure 10D**). Taken together, these results demonstrated that YPF could induce M1 macrophage polarization.

### YPF Promotes STAT1 Activation in M1 Macrophages

Macrophage polarization is a complex process including stimuli recognition and activation of the transcription factors (Biswas and Mantovani, 2010). Recent studies have shown that Signal transducers and activators of transcription 1 (STAT1) signaling pathways are involved in M1 macrophage polarization (Mosser and Edwards, 2008). To further demonstrate whether YPF induced M1 macrophage polarization, we then determined the phosphorylation of STAT1 by western blot. Result showed that YPF increased the phosphorylation of STAT1 in RAW264.7 cells in a concentration-dependent manner (**Figure 10E**). This result indicated that YPF induced M1 macrophage polarization through STAT1 signaling.

## Discussion

In this study, we have identified that YPF, an ancient Chinese herbal decoction, significantly prolonged the survival of orthotropic lung tumor-bearing mouse and increased the percentages of CD4^+^ T cells as well as their cytotoxicity. In addition, YPF increased the percentages of M1 macrophage and induced their polarization through promoting the phosphorylation of STAT1. These results demonstrated that YPF suppressed LLC cell tumor growth through inducing M1 macrophage polarization and subsequently activating CD4^+^ T cell in the tumor microenvironment.

Tumor immunity is depended on the balance between immune mediators that promote tumor progression or tumor rejection. CD4^+^ T regulatory cells, type 2 CD4^+^ T lymphocytes, type 2 natural killer T cells, myeloid-derived suppressor cells, M2 or tumor-associated macrophages, B cells, and possibly mast cells promote tumor progression, while CD8^+^ T lymphocytes, type 1 CD4^+^ T lymphocytes, natural killer, type 1 natural killer T cells, M1 macrophages, and immune killer dendritic cells promote tumor destruction (Ostrand-Rosenberg, 2008). Thus, immune cells play an important role in tumor immunosurveillance.

In the present study, we found that YPF could enhance the population of CD4^+^ T cells, but had no obvious influence in the percentage of CD8^+^ T cells in tumor tissues. In addition, YPF enhanced the cytotoxicity of CD4^+^ T cells (CD4^+^ CTLs), which depended on macrophage polarization. CD4^+^ T cells have been shown to eradicate tumors independent on CD8^+^ T cells and were more efficient in tumor rejection than CD8^+^ T cells (Perez-Diez et al., 2007). CD4 CTLs were identified as an unexpected CD4 subset with cytotoxic function by their ability to secrete granzyme B and perforin and to kill the target cells in an MHC class II-restricted fashion (Takeuchi and Saito, 2017). CD4^+^ T cells have been shown to elicit cytotoxicity and tumor rejection dependent on MHC class II-restricted recognition of tumors by tumor-reactive CD4^+^ T cells (Homma et al., 2005; Quezada et al., 2010) and participate in the anti-tumor immunity (Quezada et al., 2010; Xie et al., 2010). Our findings were consistent with these previous reports.

Tumor-associated inflammation is a hallmark of cancer (Hanahan and Weinberg, 2011). Macrophages, as immune effector cells, are the major cellular component in tumor microenvironment. They play important roles in inflammation promoting, antigen presenting, and damaged tissue remodeling (Taylor and Gordon, 2003; Jenkins et al., 2011). Macrophages also serve as an essential interface between the innate and adaptive immunity. Once being activated, they secrete a massive NO and pro-inflammatory cytokines (Chen et al., 2016a). In tumor microenvironment, macrophages upon stimulation are converted into two phenotypes entitled as M1 “classically activated” and M2 “alternatively activated” (Sica and Mantovani, 2012). In nonmalignant or regressing tumors, most of the macrophages are of the M1-like subset, representing pro-inflammatory activity, characterized by presentation antigens and promotion of tumor lysis. In contrast, macrophages in malignant tumors, generally called tumor-associated macrophages (TAMs), tend to resemble the M2-like subset, which enhance tumor growth by producing cytokines and downregulating anti-tumor immune responses (Tariq et al., 2017). To overcome the immunosuppressive and pro-tumoral functions of TAMs, current therapeutic strategies have focused on three major aspects: reduction of TAMs presence by depleting existing TAMs and/or precursors; prevention of TAMs accumulation by blocking their trafficking to the tumor site; induction of TAM reprogramming to favor antitumoral functions (Petty and Yang, 2017). The reversion of the TAM phenotype from M2 to M1 could significantly suppress the lung metastasis of Lewis tumor cells (Yuan et al., 2017). Therefore, targeting macrophages polarization is a novel therapeutic method. In the present study, we found that YPF could enhance the population of macrophages in tumor tissues and increase M1 macrophage polarization. These results suggested that YPF might be a potent regulator of M1 polarization.

Signal transducers and activators of transcription 1 (STAT1) is an important signaling molecule that is associated with M1 macrophage polarization occurring in the Th1 immune response. The phosphorylated STAT1 proteins move to the nucleus, bind specific DNA elements, and direct transcription (Darnell et al., 1994). Here, we showed that YPF increased the STAT1 phosphorylation in macrophages in a concentration-dependent manner, and enhanced the cytokines secretion, such as IL-1β and IL-12. These results were in line with previous report that STAT1 signaling in macrophages was critical for the induction of M1 macrophage activation (Wager et al., 2015).

NF-κB is a crucial factor for the regulation of both innate and adaptive immunities, controlling many genes expression when inflammatory responses occur (Chen et al., 2018). Because of the important function of NF-κB, we also detected the change of NF-κB in RAW264.7, cultured with different concentrations of YPF (data not shown). Results demonstrated that there was no obvious influence on the NF-κB signaling pathways. Taken together, YPF induced M1 macrophage polarization mainly *via* STAT1 signaling, but not NF-κB signaling.

YPF is an ancient Chinese herbal decoction, composed of three herbs. In these three herbs, Huang Qi is the most important component basing on the effect. Hence, we hypothesized Huang Qi might play the major function in regulating the tumor microenvironment. We next extracted the total saponins of astragalus (TSA), which was the primary composition of Huang Qi, then we analyzed the regulated role of TSA in tumor microenvironment. We discovered that the TSA had the similar function as YPF (data not shown). For example, TSA could significantly enhance macrophage-mediated lysis of LLC, and had no effect on CD4^+^ T cell-mediated lysis of LLC, but obviously increased CD4^+^ T cell-mediated lysis after co-incubated with macrophages. *In vitro*, we found that TSA clearly upregulated the CD107α surface expression on CD4^+^ T cells in the co-culture system. Next, we investigated whether TSA induced M1 macrophage polarization. Results showed that TSA could increase the population of CD16/32 positive macrophage cells, and mainly upregulate the expression of iNOS, IL-1β, TNF-α, and IL-12. Taken together, these results demonstrated that TSA could induce M1 macrophage polarization, and TSA might be the most important composition in YPF for regulating the tumor microenvironment. TSA is also a complex, contains several compositions. Next, we will separate the main active compound from TSA in order to further research.

In addition to the immune regulatory function of YPF, recently other studies revealed that YPF could reverse cisplatin-induced multi-drug resistance. Cisplatin (cis-diamminedichloroplatinum (II); DDP) is one of the first line treatments of chemotherapeutic drugs for NSCLC in clinic for decades. The key flaw of treating NSCLC with DDP is the development of acquired drug resistance, which enables cancer cells to evade apoptotic death, consequently leading to the reduced therapeutic efficacy (Waissbluth and Daniel, 2013; Peters and Raymond, 2016). The decreased intracellular concentration of DDP is one of the major mechanisms responsible for DDP-induced drug resistance in cancer treatment (Amable, 2016). Recently, many research results showed that co-treatment of YPF and DDP could improve the curative effects of cancers, such as hepatocarcinoma, leukopenia. Lou et al. (2016) first discovered that the reversing effect of YPF in DDP-induced resistance in NSCLC. They demonstrated that YPF increased the intracellular DDP concentration, and suppressed the expression of drug transporters. The anti-cancer effect of DDP was improved by YPF treatment with less toxicity. Furthermore, a combination of YPF and Ginkgo Folium extract, could further increase the sensitized effect on DDP-induced resistance in cultured A549/DDP cells as compared with YPF alone (Lou et al., 2018). These findings will support the prescription of traditional Chinese medicine in the cancer treatment and the application of YPF in treating NSCLC.

**Figure 11:**
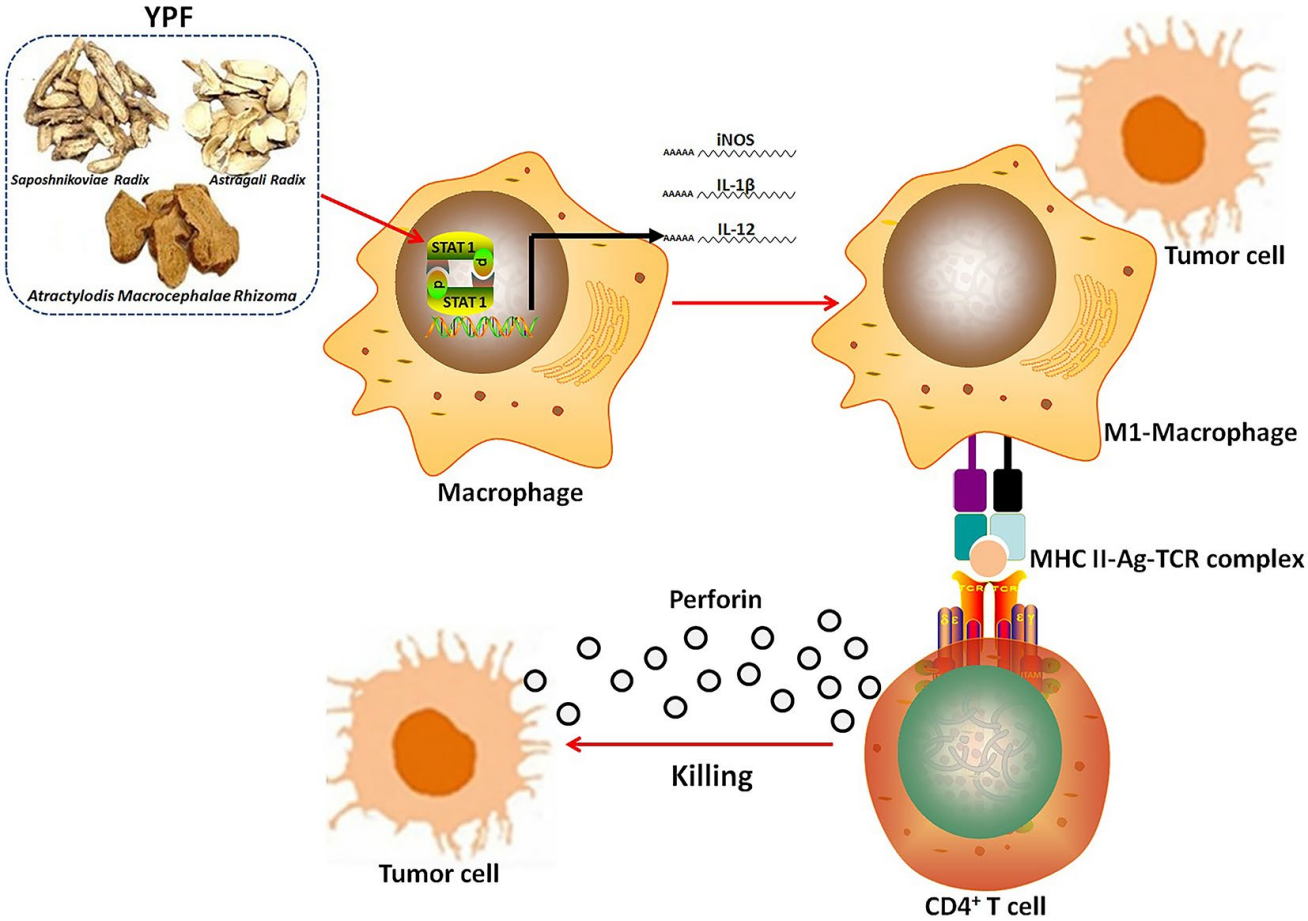
The mechanism of YPF inhibiting NSCLC. YPF can induce M1 macrophages polarization, and then activate CD4^+^ T lymphocytes, resulting in killing of LLC cells.

In summary, although YPF has been used in immune regulation for several centuries, the present study is the first study that demonstrates the inhibitory effect of YPF on NSCLC through regulating macrophages polarization to influence the tumor microenvironment. These findings provide a convince evidence that YPF can induce M1 macrophages polarization, and then activate CD4^+^ T lymphocytes, resulting in killing of LLC cells (**Figure 11**). Together all, YPF is a potent immune regulatory drug and may have a promising application in the treatment of NSCLC.

## Data Availability Statement

The raw data supporting the conclusions of this manuscript will be made available by the authors, without undue reservation, to any qualified researcher.

## Ethics Statement

All procedures related to the animal activities have been approved by Shanghai University of Traditional Chinese Medicine Institutional Animal Care and Use Committee and were conducted according to the China: Laboratory animal-Guideline for ethical review of animal welfare (GB/T 35892-2018). These guidelines were in accordance with the internationally documented principles for laboratory used and care.

## Author Contributions

LW, WW, and XZ performed the experiments and wrote the manuscript. CG, CY, ZN, XY, and CF participated in the experiments. WN performed the statistical analysis. SZ conceived the study, and participated in its design and coordination and drafted the manuscript. All authors read and approved the final manuscript.

## Funding

This study was supported by National Natural Science Foundation of China (81803933, 81903848, 81903932), Natural Science Foundation of Shanghai (19ZR1457500), Xinglin Young Talent Program of Shanghai University of Traditional Chinese Medicine, and the interdisciplinary project of Clinical Immunology of Traditional Chinese Medicine in Shanghai (30304113598). The funding body had no role in the design of the study, collection, analysis, interpretation of data, and writing the manuscript.

## Conflict of Interest

The authors declare that the research was conducted in the absence of any commercial or financial relationships that could be construed as a potential conflict of interest.
